# Subclinical atherosclerosis in rheumatoid arthritis: a case cohort analysis from ELSA-Brasil

**DOI:** 10.1590/1414-431X2025e15239

**Published:** 2026-01-30

**Authors:** P.F. Estrada, M.R.N. Cavalcante, I.S. Santos, W.R. Tebar, V. Meneghini, P.A. Lotufo, A.C. Goulart, I.M. Bensenor

**Affiliations:** 1 Centro de Pesquisa Clínica e Epidemiológica, Hospital Universitário, Universidade de São Paulo, São Paulo, SP, Brasil; 2 Departamento de Epidemiologia, Faculdade de Saúde Pública, Universidade de São Paulo, São Paulo, SP, Brasil

**Keywords:** Rheumatoid arthritis, Carotid intima-media thickness, Carotid artery plaque, Subclinical atherosclerosis, Cardiovascular risk

## Abstract

This study evaluated prevalence, incidence, and progression of carotid intima-media thickness (cIMT) and carotid artery plaques (CAP) in rheumatoid arthritis (RA) patients compared with controls during 8 years of follow-up. A case-cohort analysis of data from the ELSA-Brasil cohort was conducted, with cIMT and CAP measured by carotid ultrasound at baseline and follow-up. Linear regression was used to estimate cIMT mean and progression (ΔcIMT). Logistic regression was used to estimate odds ratios (OR) for elevated cIMT (≥75th percentile), CAP prevalence, incidence, and progression. Models were adjusted for sociodemographic and cardiovascular risk factors, excluding participants with prior cardiovascular disease. A total of 1,289 participants (188 RA, 1,101 controls) were included in the cIMT analysis and 585 (93 RA, 492 controls) in the CAP analysis. RA was not associated with baseline cIMT (β=0.00; 95%CI: -0.02-0.02; P=0.930), high cIMT (OR=1.04; 95%CI: 0.69-1.57; P=0.864), or ΔcIMT (β=0.00; 95%CI: -0.01-0.02; P=0.688). Incidence of elevated cIMT showed a non-significant trend toward higher risk in RA (OR=2.01; 95%CI: 0.88-4.59; P=0.098). No associations were found for CAP prevalence at baseline (OR=1.64; 95%CI: 0.92-2.91; P=0.090), prevalence at follow-up (OR=0.75; 95%CI: 0.41-1.36; P=0.342), incidence (OR=0.78; 95%CI: 0.37-1.63; P=0.508), or progression (β=-0.33; 95%CI: -0.72-0.07; P=0.102). This study found no independent association between RA and cIMT or CAP.

## Introduction

Rheumatoid arthritis (RA) is projected to affect 31.7 million individuals worldwide by 2050, according to the Global Burden of Disease Study, underscoring its growing public health relevance (1). In addition to its debilitating joint manifestations, RA is characterized by systemic inflammation that accelerates vascular injury and contributes to increased cardiovascular mortality (2,3). Although the articular effects of RA are well recognized, its systemic consequences are equally important, particularly in promoting accelerated atherosclerosis and cardiovascular disease (CVD), which remains the leading cause of death among individuals with RA (4,5). Beyond traditional cardiovascular risk factors, RA itself is considered an independent risk factor for atherosclerosis, likely mediated by chronic immune activation, endothelial dysfunction, and metabolic disturbances (4).

Subclinical atherosclerosis, commonly identified through noninvasive vascular imaging, plays a critical role in the early detection of cardiovascular risk. Carotid intima-media thickness (cIMT) and the presence of carotid artery plaques (CAP) are widely accepted surrogate markers of subclinical atherosclerosis and have been shown to predict future cardiovascular events in both the general population and individuals with RA (6-8).

Previous studies have demonstrated that individuals with RA exhibit increased cIMT and a higher prevalence of CAP (6-12). However, most of the available evidence originates from high-income countries, often based on relatively homogeneous cohorts from North America and Europe (9-11,13,14). Furthermore, most studies have limitations related to cross-sectional design and/or follow-up duration, restricting inferences about the prevalence, incidence, and progression of atherosclerosis (6,11,14).

Therefore, this study aimed to evaluate the association of RA with the prevalence, incidence, and progression of subclinical atherosclerosis, assessed through cIMT and CAP, over an 8-year follow-up period. The analysis was conducted using data from the Brazilian Longitudinal Study of Adult Health (ELSA-Brasil) in a case-cohort design restricted to participants without prior cardiovascular disease.

## Material and Methods

### Study design and population

This study employed a case cohort design within the ELSA-Brasil study, a prospective multicenter cohort of 15,105 civil servants aged 35 to 74 years, recruited from six Brazilian cities between 2008 and 2010. The primary objective of ELSA-Brasil was to investigate risk factors for CVD and diabetes (15,16). A notable feature of the study is its ability to assess not only classical risk factors for CVD but also nontraditional ones, including chronic inflammatory conditions such as RA (17).

The present analysis used baseline data (2008-2010) and 8-year follow-up data (2017-2019) to evaluate the association between RA and markers of subclinical carotid atherosclerosis. The study design enabled efficient analysis of cohort data by using detailed measurements from a subsample while preserving analytical validity (18).

cIMT was measured at baseline and again at follow-up in the entire cohort. CAP was assessed at both time points, but only at the São Paulo site and in participants with standardized bilateral carotid ultrasound images. All ultrasound procedures were conducted by trained technicians using standardized protocols under strict quality control (19,20).

The study protocol was approved by the Research Ethics Committees at all six participating centers (Universidade Federal da Bahia - UFBA: 027/06; Universidade Federal de Minas Gerais - UFMG: 186/06; Universidade Federal do Espírito Santo - UFES: 041/06; Fundação Oswaldo Cruz - FIOCRUZ: 343/06; Universidade de São Paulo - USP: 669/06; and Universidade Federal do Rio Grande do Sul - UFRGS: 194/06). Written informed consent was obtained from all participants prior to enrollment.

### Chronic inflammatory diseases and definition of RA

During the baseline examination (2008-2010), RA status was determined based on self-report of a physician diagnosis, using the question: “Have you ever been told by a doctor that you have or had rheumatoid arthritis?” (17). Additional information was collected on the use of disease-modifying antirheumatic drugs (DMARDs) within 14 days prior to data collection. DMARDs included both conventional and biologic agents, such as hydroxychloroquine, methotrexate, leflunomide, sulfasalazine, azathioprine, cyclosporine, cyclophosphamide, tumor necrosis factor inhibitors, and interleukin antagonists (17).

The analytic definition of RA used in this study was consistent with previous population-based studies, including the Women's Health Initiative (WHI) study (21).

Among the 15,105 ELSA-Brasil participants, a high-risk group for inflammatory diseases was identified based on at least one of the following criteria: self-reported physician diagnosis of RA, current use of DMARDs, or persistently elevated high-sensitivity C-reactive protein (hs-CRP), measured using an immunochemical assay in a nephelometric analyzer (Siemens BN II System, Germany). Individuals meeting any of these criteria were enrolled in the Chronic Inflammatory Diseases (CID) ancillary study and underwent further biomarker assessment, including rheumatoid factor (RF Latex, WAMA, Brazil) and anti-cyclic citrullinated peptide antibodies (anti-CCP IgG-ELISA, Euroimmun, Germany). RA was confirmed when at least two of the following were present: prior medical diagnosis of RA, use of DMARDs, and seropositivity for anti-CCP or RF (17).

### Control group

ELSA-Brasil was designed to perform case cohort studies. The Aleatory Cohort Sample (ACS), comprising roughly 10% of the entire ELSA-Brasil baseline cohort (n=1543 of 15,105 participants), was randomly selected at study inception to be representative of the full cohort. Participants in the ACS underwent the same standardized interviews and clinical, biochemical, and imaging examinations as the rest of the cohort, with additional biological samples collected and stored at each visit. The ACS served as a fixed reference group for ancillary studies and maintained the cohort characteristics while having lower costs and logistical demands compared with testing the entire cohort (17).

Within this case cohort design, all confirmed RA cases in ELSA-Brasil were compared against the ACS participants, enabling unbiased estimation of associations between RA and subclinical atherosclerosis while maintaining efficiency in resource utilization (17).

### Study participants

**Figure 1:**
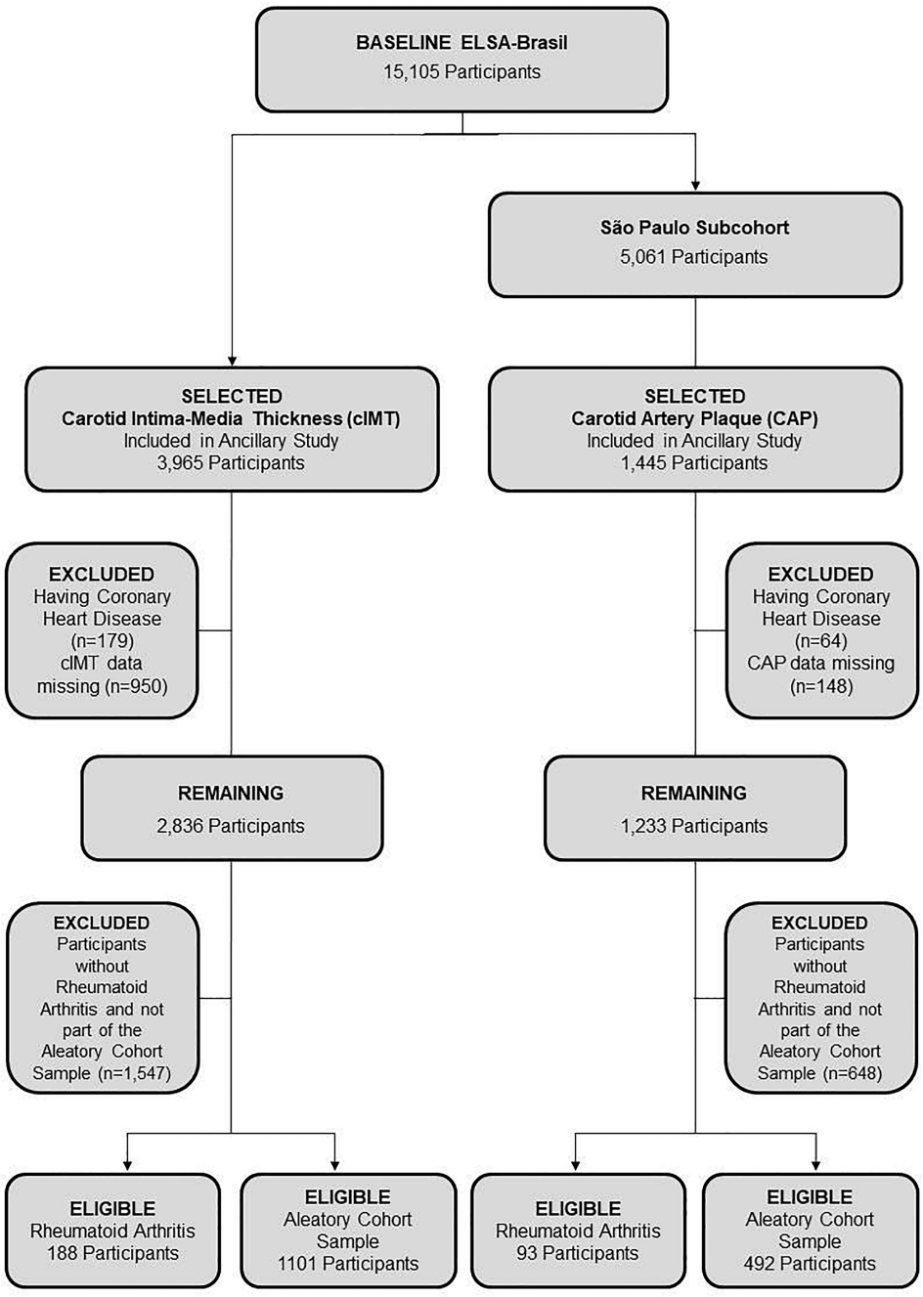
Flowchart detailing participant selection for carotid intima-media thickness (cIMT) and carotid artery plaque (CAP) analyses.

For the cIMT analysis, 3,965 individuals from the CID ancillary study were initially considered. After excluding those with prior coronary heart disease (n=179) and those without cIMT data (n=950), 2,836 participants remained. Subsequently, 1,547 individuals who neither had RA nor were part of the ACS were excluded. The final analytical sample for the cIMT analysis comprised 188 participants with RA and 1,101 controls from the ACS (Figure 1).

For the CAP analysis, only participants from the São Paulo Research Center (n=5,061) were eligible, because CAP data were exclusively available at this site. Among these, 1,445 individuals were included in the CID ancillary study. After excluding participants with prior coronary heart disease (n=64) and those with missing CAP data (n=148), 1,233 remained. Of these, 648 individuals without RA and not part of the ACS were excluded, resulting in a final analytical sample of 93 participants with RA and 492 controls (Figure 1).

### Clinical variables

Clinical data were obtained at baseline through standardized questionnaires, physical examinations, and laboratory analyses. Medication use during the 14 days prior to the baseline visit was recorded (15). Diabetes was defined as a self-reported physician diagnosis, current use of antidiabetic medications, or meeting laboratory criteria: fasting plasma glucose ≥126 mg/dL, random plasma glucose ≥200 mg/dL, or glycated hemoglobin (HbA1c) ≥6.5% (22). Hypertension was defined as systolic blood pressure ≥140 mmHg and/or diastolic blood pressure ≥90 mmHg or the use of antihypertensive medications (23).

Dyslipidemia was defined as low-density lipoprotein cholesterol (LDL-C) ≥130 mg/dL or the use of lipid-lowering therapy. Lipid fractions were measured using enzymatic colorimetric assays. LDL-C was calculated using the Friedewald equation unless triglyceride levels exceeded 400 mg/dL, in which case a direct measurement was used. Body mass index (BMI) and waist circumference were measured using standardized techniques (24). GlycA concentrations were quantified by nuclear magnetic resonance spectroscopy (LipoProfile 4, LabCorp, USA) and reported in micromoles per liter (μmol/L). GlycA is a validated biomarker of systemic inflammation with low analytical variability (25).

### Sociodemographic variables

Sociodemographic and health-related information was obtained using structured interviews. Sex was recorded as male or female and treated as a bivariate variable. Age was collected as a continuous variable and categorized into four groups: 35-44, 45-54, 55-64, and 65-74 years (16).

Race/ethnicity was self-reported according to categories established by the Brazilian national census: White, Black, Mixed, Asian, and Indigenous. These classifications reflect the sociocultural structure of Brazil. Race and ethnicity were capitalized, not abbreviated, and reported descriptively in accordance with current guidelines (16).

Educational level was categorized as: less than high school, high school or some college, and college graduate or higher. Family income was categorized in U.S. dollars based on a 2:1 exchange rate at baseline: <1,245, 1,246-$3,319, and ≥$3,320 (16).

### Carotid intima-media thickness (cIMT)

Carotid ultrasound scans were acquired using a Toshiba Aplio XG device (Japan) with a 7.5 MHz linear transducer. Image quality was verified by trained technicians at the Central Reading Center in São Paulo. Scans were recorded over three cardiac cycles following the recommendations of the American and Brazilian Societies of Echocardiography (26). The region of interest (ROI) for cIMT measurement was defined as a 1-cm segment of the far wall of the common carotid artery, located 1 cm below the carotid bulb. This location was used consistently, regardless of plaque presence, to reduce measurement variability and subjective bias. cIMT was assessed using MIA software (USA), a semi-automated tool that identifies the lumen-intima and media-adventitia interfaces across frames, providing mean cIMT values without manual measurement. Analyses were conducted by board-certified technicians blinded to clinical data, following a standardized protocol (26).

For each participant, cIMT values were averaged across left and right carotid arteries at baseline and at the 8-year follow-up. Detailed methodology for cIMT assessment in ELSA-Brasil has been published previously (27).

### Carotid artery plaque (CAP)

Carotid ultrasound was performed using a B-mode ultrasound device (Toshiba Aplio XG) equipped with a 7.5 MHz linear transducer. Images were acquired transversely and longitudinally from the common carotid artery, carotid bulb, and internal carotid origin, following the Mannheim Consensus Statement (28). These anatomical sites have been consistently used in major atherosclerosis studies such as the Multi-Ethnic Study of Atherosclerosis (MESA) (29). CAP was defined per the Mannheim Consensus, as a focal structure encroaching into the arterial lumen by ≥0.5 mm or ≥50% of the surrounding IMT value, or a wall thickness >1.5 mm measured from the media-adventitia to the intima-lumen interface (28). Ultrasound images were centrally analyzed at the ELSA-Brasil Core Laboratory in São Paulo to ensure methodological consistency across participants (30). Trained radiologists evaluated the presence of plaques on both near and far walls bilaterally across 12 segments. Each segment with plaque was assigned 1 point, and the total plaque score ranged from 0 to 12. Circumferential plaques involving near and far walls in the same segment scored 2 points. The scoring system demonstrated excellent reproducibility, with intra- and inter-observer kappa values of 0.96 and 0.99, respectively (30).

For this study, plaque burden was categorized semi-quantitatively (0, 1, ≥2). Incident plaque was defined as the appearance of at least one new plaque in those with no baseline plaque. Progression was assessed by changes in plaque score categories over the 8-year follow-up.

### Statistical analysis

Descriptive statistics were used to summarize baseline characteristics. Continuous variables are reported as means±SD or median (IQR), and categorical variables as counts and percentages. Group comparisons (RA *vs* controls) were performed with independent *t*-tests or Mann-Whitney U tests for continuous variables and chi-squared tests for categorical variables; normality was assessed with the Shapiro-Wilk test.

Linear regression was used to estimate differences in mean cIMT at baseline and absolute progression over 8 years (ΔcIMT). Logistic regression was used to evaluate the odds of elevated cIMT, defined as values at or above the 75th percentile of the cohort distribution at baseline (prevalence) and at follow-up (incidence).

Logistic regression models were used to assess associations between RA and plaque presence at baseline (Wave 1) and at follow-up (Wave 3), and the incidence of new plaques over 8 years among participants without baseline plaques. Linear regression was used to evaluate CAP progression, defined as the change in total plaque score from Wave 1 to Wave 3.

All regression models followed a hierarchical adjustment strategy: Model 1 included age, sex, race, and education; Model 2 additionally included diabetes and hypertension; and Model 3 further adjusted for dyslipidemia. Results are reported as β coefficients or odds ratios (OR), with corresponding 95% confidence intervals (CI) and P-values. All statistical tests were two-sided, and P-values <0.05 were considered statistically significant. Analyses were performed using R statistical software, version 4.5.1 (R Foundation for Statistical Computing, Austria, 2025).

## Results

At baseline, individuals with RA were older than controls in both the cIMT (median age: 54 *vs* 50 years, P=0.001) and CAP (54 *vs* 50 years, P=0.023) subsamples. As expected, the RA group was predominantly female (77.1 *vs* 55.3% for cIMT and 82.7 *vs* 51.2% for CAP; both P<0.001). Compared with controls, participants with RA had significantly higher levels of hsCRP (P<0.001 for cIMT and P=0.043 for CAP), higher GlycA concentrations (both P<0.01), and lower triglyceride levels in the CAP sample (median: 91 *vs* 110 mg/dL, P=0.022) (Tables 1 and 2).

**Table 1:** Baseline characteristics of individuals with rheumatoid arthritis (RA) and controls from the aleatory cohort sample (ACS) included in the carotid intima-media thickness (cIMT) analysis.

Variables	RA (n=188)	ACS (n=1101)	P-value
Age (years), median (IQR)	54 (47-61)	50 (45-57)	**0.001**
Female sex, n (%)	145 (77.1%)	609 (55.3%)	**<0.001**
Race/Ethnicity, n (%)			0.398
White	104 (55.9%)	583 (53.3%)	
Mixed	38 (20.4%)	284 (26.0%)	
Black	34 (18.2%)	168 (15.3%)	
Asian	6 (3.2%)	43 (3.9%)	
Indigenous	4 (2.1%)	14 (1.2%)	
Education, n (%)			0.250
Less than high school	25 (13.2%)	127 (11.5%)	
High school/some college	72 (38.2%)	369 (33.5%)	
College or more	91 (48.4%)	605 (54.9%)	
Family income (USD), n (%)			0.028
<1,245	51 (27.1%)	266 (24.2%)	
1,245-3,319	95 (50.5%)	481 (43.7%)	
>3,320	42 (22.3%)	352 (32.0%)	
BMI (kg/m^2^), median (IQR)	26.4 (23.6-30.2)	26.0 (23.6-29.0)	0.101
Waist circumference (cm), median (IQR)	89.2 (80.5-99.1)	89.7 (80.8-97.3)	0.974
Hypertension, n (%)	64 (34.2%)	329 (29.9%)	0.271
Diabetes, n (%)	31 (16.4%)	158 (14.3%)	0.512
Dyslipidemia, n (%)	80 (42.7%)	480 (43.6%)	0.881
Total cholesterol (mg/dL), mean±SD	196.9±40.3	200.4±40.8	0.495
HDL cholesterol (mg/dL), mean±SD	54.9±14.4	54.5±13.3	0.793
LDL cholesterol (mg/dL), mean±SD	115.3±33.8	118.6±35.4	0.301
Triglycerides (mg/dL), median (IQR)	99 (73-149)	105 (77-149)	0.296
C-reactive protein (mg/L), median (IQR)	2.1 (1.0-5.0)	1.3 (0.7-3.0)	**<0.001**
GlycA, median (IQR)	436.5 (390-478)	407.0 (367-450)	**<0.001**
cIMT, mm, Wave 1, mean±SD	0.6±0.1	0.6±0.1	0.145
cIMT, mm, Wave 3, mean±SD	0.7±0.1	0.7±0.1	0.691
cIMT ≥75th percentile, Wave 1, n (%)	54 (28.7%)	272 (24.7%)	0.279
cIMT ≥75th percentile, Wave 3, n (%)	37 (24.2%)	202 (25.4%)	0.794
Incidence of cIMT ≥ 75th percentile, n (%)	7 (6.3%)	72 (11.7%)	0.125

Continuous variables are reported as median (interquartile range-IQR) or means±SD according to data distribution. Normality was assessed before group comparisons. Independent *t*-tests or Mann-Whitney U tests were used for continuous variables, and chi-squared tests for categorical variables. LDL: low-density lipoprotein; HDL: high-density lipoprotein.

**Table 2:** Baseline characteristics of individuals with rheumatoid arthritis (RA) and controls from the aleatory cohort sample (ACS) included in the carotid artery plaque (CAP) analysis.

Variables	RA (n=93)	ACS (n=492)	P-value
Age (years), median (IQR)	54 (48-60)	50 (46-57)	**0.023**
Female sex, n (%)	77 (82.7%)	252 (51.2%)	**<0.001**
Race/Ethnicity, n (%)			0.628
White	52 (56.5%)	287 (58.9%)	
Mixed	16 (17.3%)	98 (20.1%)	
Black	18 (19.5%)	65 (13.3%)	
Asian	5 (5.4%)	30 (6.1%)	
Indigenous	1 (1.0%)	7 (1.4%)	
Education, n (%)			0.288
Less than high school	17 (18.2%)	65 (13.2%)	
High school/some college	38 (40.8%)	189 (38.4%)	
College or more	38 (40.8%)	238 (48.3%)	
Family income (USD), n (%)			0.093
<1,245	33 (35.4%)	133 (27.1%)	
1,245-3,319	40 (43.0%)	201 (41.0%)	
>3,320	20 (21.5%)	156 (31.8%)	
BMI (kg/m^2^), median (IQR)	26.0 (23.6-30.9)	26.4 (23.8-29.4)	0.745
Waist circumference (cm), median (IQR)	86.6 (78.5-97.0)	89.8 (80.8-97.1)	0.212
Hypertension, n (%)	33 (35.4%)	152 (30.8%)	0.452
Diabetes, n (%)	15 (16.1%)	79 (16.0%)	0.990
Dyslipidemia, n (%)	33 (35.4%)	216 (43.9%)	0.159
Total cholesterol (mg/dL), mean±SD	192.2±37.6	200.9±42.7	0.066
HDL cholesterol (mg/dL), mean±SD	54.7±14.5	54.0±12.9	0.904
LDL cholesterol (mg/dL), mean±SD	112.8±32.5	118.9±35.7	0.085
Triglycerides (mg/dL), median (IQR)	91 (69-139)	110 (79-147)	**0.022**
C-reactive protein (mg/L), median (IQR)	1.9 (0.89-5.52)	1.5 (0.74-3.42)	**0.043**
GlycA, median (IQR)	436.5 (387-478)	408.0 (368-456)	**0.008**
Plaque Wave 1, n (%)	28 (30.1%)	186 (37.8%)	0.195
Plaque Wave 3, n (%)	33 (49.2%)	129 (43.7%)	0.493
Incidence of plaque, n (%)	16 (34.0%)	61 (31.7%)	0.900

Continuous variables are reported as median (interquartile range-IQR) or means±SD, according to data distribution. Normality was assessed before group comparisons. Independent t-tests or Mann-Whitney U tests were used for continuous variables, and chi-squared tests for categorical variables. Plaque refers to the presence of carotid artery plaque, defined according to Mannheim Criteria as focal thickening >0.5 mm or >50% relative to adjacent cIMT. LDL: low-density lipoprotein; HDL: high-density lipoprotein.

Regarding cIMT, no statistically significant differences were observed between groups. RA was not associated with baseline mean cIMT (β=0.0007, 95%CI: -0.0155 to 0.0170, P=0.930), prevalence of elevated cIMT (≥75th percentile) at baseline (OR=1.04, 95%CI: 0.69-1.57, P=0.864), or incidence of elevated cIMT at follow-up (OR=2.01, 95%CI: 0.88-4.59, P=0.098) (Table 3). In the 8-year progression analysis, no association was found between RA and change in cIMT over time (β=0.0036, 95%CI: -0.0139 to 0.0210, P=0.688) (Table 3).

**Table 3:** Linear (β coefficients) and logistic regression analyses (odds ratio and 95%CI) comparing participants with rheumatoid arthritis and controls: baseline mean carotid intima-media thickness (cIMT) prevalence, elevated cIMT ≥75th percentile at follow-up incidence, and 8-year ΔcIMT progression.

Analysis	Model	β/OR	95%CI	P-value
Prevalence (mean cIMT)	Unadjusted	-0.0129	-0.0320-0.0061	0.183
	Model 1	-0.0001	-0.0166-0.0164	0.992
	Model 2	0.0009	-0.0153-0.0172	0.909
	Model 3	0.0007	-0.0155-0.0170	0.930
Prevalence (cIMT ≥75th pctl)	Unadjusted	0.8142	0.5770-1.1489	0.242
	Model 1	1.0135	0.6783-1.5143	0.947
	Model 2	1.0374	0.6901-1.5595	0.859
	Model 3	1.0367	0.6862-1.5664	0.864
Incidence (cIMT ≥75th pctl)	Unadjusted	1.9810	0.8867-4.4257	0.095
	Model 1	2.0406	0.8994-4.6298	0.088
	Model 2	2.0792	0.9125-4.7373	0.081
	Model 3	2.0095	0.8792-4.5926	0.098
Progression (ΔcIMT, 8 years)	Unadjusted	0.0074	−0.0097-0.0245	0.395
	Model 1	0.0036	−0.0137-0.0210	0.682
	Model 2	0.0037	−0.0137-0.0212	0.674
	Model 3	0.0036	−0.0139-0.0210	0.688

Models were progressively adjusted. Crude model: unadjusted; Model 1: adjusted for age, sex, race, and education; Model 2: additionally adjusted for diabetes and hypertension; Model 3: additionally adjusted for dyslipidemia. pctl: percentile.

A non-significant trend toward a higher prevalence of CAP at baseline was found among individuals with RA (OR=1.64, 95%CI: 0.92-2.91, P=0.090). However, no significant associations were found for CAP prevalence at follow-up (OR=0.75, 95%CI: 0.41-1.36, P=0.342) (Table 4). Similarly, the incidence of new plaques between baseline and follow-up did not differ between groups (OR=0.78, 95%CI: 0.37-1.63, P=0.508). In the 8-year progression analysis, RA was not associated with change in total plaque score (β=-0.2997, 95%CI: -0.7061 to 0.1067, P=0.148) (Table 4).

**Table 4:** Linear (β coefficients) and logistic regression analyses (odds ratio and 95%CI) comparing participants with rheumatoid arthritis and controls: carotid plaque (CAP) prevalence at Wave 1 and Wave 3, CAP incidence over 8 years, and CAP progression.

Analysis	Model	OR/β	95%CI	P-value
Prevalence (Wave 1)	Unadjusted	1.4111	0.8740-2.2782	0.158
	Model 1	1.6767	0.9466-2.9700	0.076
	Model 2	1.6843	0.9496-2.9874	0.074
	Model 3	1.6394	0.9247-2.9064	0.090
Prevalence (Wave 3)	Unadjusted	0.8007	0.4706-1.3621	0.412
	Model 1	0.7367	0.4106-1.3216	0.305
	Model 2	0.7626	0.4215-1.3797	0.370
	Model 3	0.7496	0.4135-1.3586	0.342
Incidence of CAP (8 years)	Unadjusted	0.9022	0.4591-1.7728	0.765
	Model 1	0.7928	0.3828-1.6421	0.532
	Model 2	0.7785	0.3721-1.6284	0.506
	Model 3	0.7793	0.3724-1.6310	0.508
Progression (8 years)	Unadjusted	−0.3276	−0.7203-0.0651	0.102
	Model 1	−0.3430	−0.7504-0.0645	0.099
	Model 2	−0.3204	−0.7282-0.0873	0.123
	Model 3	−0.2997	−0.7061-0.1067	0.148

Models were progressively adjusted. Crude model: unadjusted; Model 1: adjusted for age, sex, race, and education; Model 2: additionally adjusted for diabetes and hypertension; Model 3: additionally adjusted for dyslipidemia.

## Discussion

In this cohort study with eight years of follow-up, no statistically significant association was found between RA and cIMT or CAP. Although individuals with RA exhibited higher baseline levels of systemic inflammatory markers (hs-CRP and GlycA), RA was not independently associated with mean cIMT or with prevalence, incidence, or progression of elevated cIMT. Similarly, no association was observed between RA and prevalence, incidence, or progression of CAP after multivariate adjustment.

The analytical strategy for cIMT progression evaluation was based on a previously validated model within the ELSA-Brasil cohort. A prior study applying the same framework reported no association between psoriasis and cIMT progression over an 8-year follow-up (31), supporting the robustness of the model. Our findings are also consistent with other studies that did not find significant associations between RA and markers of subclinical atherosclerosis. One cross-sectional study involving 243 patients with clinically controlled RA found similar cIMT values between cases and controls, with hypertension identified as the only independent predictor of increased cIMT (32). Likewise, a longitudinal analysis with 139 matched pairs reported no difference in cIMT progression after adjusting for cardiovascular risk factors and medication use (33).

Sex distribution may also have influenced our findings. Both the cIMT and CAP groups had a predominance of women with RA (77.1 and 82.7%, respectively), consistent with the known higher prevalence of RA among females. In general, women tend to access healthcare services more frequently and participate more in preventive care than men. These behaviors could lead to earlier diagnosis, more consistent disease management, and reduced variability in subclinical atherosclerosis markers (34).

A recent systematic review and meta-analysis including over 165,000 individuals demonstrated that men have consistently higher mean cIMT values across carotid segments, with the age-related increase occurring approximately 10 years later in women. The predominance of women in our RA group may therefore have attenuated differences in subclinical atherosclerosis between RA and controls. As the ACS was designed to represent the entire cohort by randomization, some differences with the RA group were expected, reinforcing the need to adjust for sex and other potential confounders in the regression models (35).

Regarding CAP, our results diverge from previous studies that identified RA as a predictor of plaque development. A systematic review and meta-analysis including 35 studies reported higher odds of carotid plaque in RA compared to controls (6). A six-year prospective study excluding participants with classic cardiovascular risk factors also found increased odds of incident CAP among RA patients with moderate or high disease activity (9). In contrast, the TOMORROW study found no significant difference in new CAP incidence between RA patients and matched controls, and RA was not an independent predictor of plaque formation (36).

These discrepancies likely reflect differences in inflammatory burden, treatment exposures, and cohort characteristics. In the ELSA-Brasil, the absence of association may be attributable to the relatively low disease severity and the consistent access to medical care within an occupational cohort. Moreover, the prognostic relevance of CAP is underscored by evidence demonstrating that its presence independently predicts future cardiovascular events in RA populations (11), reinforcing its clinical importance regardless of its association with RA status.

The absence of association observed in our study must be interpreted considering prior findings that link chronic inflammation in RA to subclinical atherosclerosis. Previous studies have reported associations between elevated disease activity and both cIMT progression and CAP formation (6,9-11). These associations appear more pronounced in populations with sustained inflammatory activity or limited access to immunomodulatory therapies. The relatively low inflammatory activity in our cohort may explain the null findings. ELSA-Brasil participants were predominantly employed and had broad access to healthcare, including high-cost medications, and a previous study showed that cardiovascular risk in RA is linked to sustained systemic inflammation, with biological therapies reducing this risk (37). Although the use of DMARDs was documented, information on treatment duration, cumulative exposure, and therapeutic intensity was unavailable. Prior studies suggest that methotrexate and biologic agents may reduce cardiovascular risk by modulating systemic inflammation and endothelial function (38,39). Therefore, it is possible that the absence of association in our study was influenced, at least in part, by DMARDs use. On the other hand, inflammatory biomarkers were higher in the patients with RA compared to controls, suggesting inflammatory activity in the RA group. The inclusion of a lower-risk population (compared to those in clinical settings) can also explain these findings.

This study has notable methodological strengths, including standardized definitions of RA based on clinical criteria, medication use, and serological markers, which minimize misclassification bias. Data collection followed harmonized protocols across clinical, laboratory, and imaging domains. The multicenter design and inclusion of participants from six urban centers with diverse racial and socioeconomic backgrounds increase the external validity of the findings.

Nevertheless, some limitations must be acknowledged. Despite statistical adjustments, residual confounding cannot be excluded. The cohort is composed mainly of employed individuals with higher average income than the general population, which may underrepresent individuals with severe or poorly controlled RA. The relatively young median baseline age of approximately 50 years may have limited the occurrence of subclinical atherosclerosis events during follow-up. Further longitudinal analyses with extended follow-up and detailed treatment data are warranted. In conclusion, RA was not associated with cIMT or CAP burden over an eight-year period in this cohort. The prevalence, incidence, and progression of subclinical carotid atherosclerosis did not differ significantly between individuals with and without RA, after adjusting for traditional cardiovascular risk factors.

## Data Availability

All data generated or analyzed during this study are included in this published article.
